# Drug Resistance Mechanisms of *Mycoplasma pneumoniae* to Macrolide Antibiotics

**DOI:** 10.1155/2014/320801

**Published:** 2014-01-28

**Authors:** Xijie Liu, Yue Jiang, Xiaogeng Chen, Jing Li, Dawei Shi, Deli Xin

**Affiliations:** ^1^Pediatric Apartment, Beijing Friendship Hospital, Capital Medical University, Beijing 100050, China; ^2^Beijing Institue of Tropical Medicine, Beijing Friendship Hospital, Capital Medical University, No. 95 Yongan Road, Xicheng District, Beijing 100050, China

## Abstract

Throat swabs from children with suspected *Mycoplasma pneumoniae* (*M. pneumoniae*) infection were cultured for the presence of *M. pneumoniae* and its species specificity using the 16S rRNA gene. Seventy-six *M. pneumoniae* strains isolated from 580 swabs showed that 70 were erythromycin resistant with minimum inhibitory concentrations (MIC) around 32–512 mg/L. Fifty *M. pneumoniae* strains (46 resistant, 4 sensitive) were tested for sensitivity to tetracycline, ciprofloxacin, and gentamicin. Tetracycline and ciprofloxacin had some effect, and gentamicin had an effect on the majority of *M. pneumoniae* strains. Domains II and V of the 23S rRNA gene and the ribosomal protein L4 and L22 genes, both of which are considered to be associated with macrolide resistance, were sequenced and the sequences were compared with the corresponding sequences in M129 registered with NCBI and the FH strain. The 70 resistant strains all showed a 2063 or 2064 site mutation in domain V of the 23S rRNA but no mutations in domain II. Site mutations of L4 or L22 can be observed in either resistant or sensitive strains, although it is not known whether this is associated with drug resistance.

## 1. Introduction

*Mycoplasma pneumoniae* is a common pathogen of respiratory tract infection in children and adolescents and can cause serious pneumonia and external lung complications [1]. At present, the preferred treatment is macrolide antibiotics. In recent years, however, many countries have reported the isolation of clinically drug-resistant strains [2–5], the main mechanism of resistance being a mutation in the 23S rRNA gene which is the target of macrolide antibiotic action [6, 7]. In China, there are a number of reports of clinically resistant *M. pneumoniae* strains [8, 9], resulting in serious failure of effective antibacterial agents. In this study macrolide-resistant *M. pneumoniae* strains isolated between 2003 and 2007 were screened for sensitivity to antibacterial activity of tetracycline, ciprofloxacin, and gentamicin. The strains were typed for domain II and V of 23S rRNA, and for the ribosomal protein L4 and L22 genes, which are also associated with macrolide resistance.

## 2. Materials and Methods

### 2.1. Isolation, Culture, and Identification of *M. pneumoniae*

#### 2.1.1. Specimens

580 cases of throat swab specimens were collected from the suspected *M. pneumoniae* infected children (age range: 1–14) in the pediatric wards of Beijing Friendship Hospital affiliated to the Capital University of Medical Sciences.

#### 2.1.2. *M. pneumoniae* Culture Medium

PPLO basic medium was used containing 15% newborn calf serum, 10% fresh yeast extract, 0.4% phenol red indicator, 1% glucose, and 50000 U/100 mL penicillin.

#### 2.1.3. Culture and Identification

The throat swab specimens were inoculated in *M. pneumoniae* liquid medium, mixed evenly, and placed in an incubator at 37°C, with 5% CO_2_ for cultivation. Growth of *M. pneumoniae* causes decrease of PH of the medium, which is indicated by a color change (from red to yellow) in the indicator. Nested PCR for species-specific *M. pneumoniae* identification was carried out on all positive samples using primers for the 16S rRNA gene [2].

### 2.2. *In Vitro* Drug Sensitivity Test

Equal volumes of *M. pneumoniae* bacteria fluid (concentration: 10^5^ CCU/mL) and drug (diluted to the desired concentrations with *M. pneumoniae* medium) were incubated in an incubator at 37°C, with 5% CO_2_. Color change of the medium was used to estimate the minimum drug concentration that could inhibit *M. pneumoniae* growth (MIC). Erythromycin, tetracycline, ciprofloxacin, and gentamicin were all purchased from the National Institute for the Control of Pharmaceutical and Biological Products.

### 2.3. Analysis of Domains II and V of 23S rRNA Gene and the Ribosomal Protein L4 and L22 Genes

#### 2.3.1. DNA Extraction

20 *μ*L *M. pneumoniae* bacteria fluid was collected, centrifuged at 12000 r/min for 10 min, and the supernatant was removed. 200 *μ*L 1% Triton X-100 was added to the pellet, mixed evenly, and placed in a boiling water bath for 10 min.

#### 2.3.2. PCR Amplification and DNA Sequencing

The primers for amplification of a 793 fragment of domain V of the 23S rRNA gene were designed (P3 5′ TAACTATAACGGTCCTAAGG 3′ and P4 5′ CGCTACAACTGGAGCATAAGA 3′); primers for domain II of 23S rRNA and for L4 and L22 were as reported by Waites and Talkington [1] (see Table 1). The PCR mixture for domain II of 23S rRNA with L4 and L22 contained 15 pmol in each of forward and reverse primers, 1U TaqDNA polymerase, 20 *μ*L total reaction volume, and 5 *μ*L DNA template. PCR conditions were initial denaturation for 2 min at 94°C, followed by 94°C for 45 s, 55°C for 1 min, 72°C for 30 cycles, and a final 5 min extension cycle at 72°C. The specific nested PCR method for domain V of 23S rRNA was as described in [2]. The amplified products were purified and subjected to full-auto DNA sequencing (ABI 3730XL sequenator from Shanghai Sangon Biological Technologies & Service Co., Ltd.). Resulting sequences were compared with the corresponding sequences of the standard strain M129 registered at NCBI.

## 3. Results

### 3.1. *M. pneumoniae* Culture and Identification

76 *M. pneumoniae* strains were isolated from 580 cases of throat swab specimens, and all 76 strains and the FH strain showed the same species-specific 417 bp *M. pneumoniae* fragment of the 16S rRNA gene typical of the species.

### 3.2. Antibiotic Sensitivity Tests

#### 3.2.1. Erythromycin MIC

Among the 76 *M. pneumoniae* strains, 70 showed erythromycin resistance, with a significantly higher erythromycin MIC around 32–512 mg/L.

#### 3.2.2. MICs of Tetracycline, Ciprofloxacin, and Gentamicin

The *in vitro* drug sensitivity tests of these three drugs were performed on 50 strains, 46 of which were erythromycin resistant and 4 erythromycin sensitive.

*The Tetracycline MIC of the Standard Strain FH Was 2 mg/L*. The MICs of the 50 isolated *M. pneumoniae* strains was between 0.5 and 2 mg/L, all within the sensitive range.

*The Ciprofloxacin MIC of the Standard Strain FH Was 1 mg/L*. The MICs of the 50 isolated *M. pneumoniae* strains was between 1 and 2 mg/L, most being within the sensitive range, only 5 *M. pneumoniae* strains showing MICs slightly higher than that of the standard strain.

*The Gentamicin MIC of the Standard Strain FH Was 4 mg/L*. The MICs of the 50 isolated strains was between 2 and 16 mg/L, 33 samples being within the sensitive range, the remaining 17 being higher than that of the standard strain.

The categorization of sensitivity or resistance according to the MIC values of erythromycin, tetracycline, ciprofloxacin, and gentamicin follows the standard formulated by NCCLS in 2006 [10].

### 3.3. PCR Amplification and DNA Sequencing

**Figure 1:**
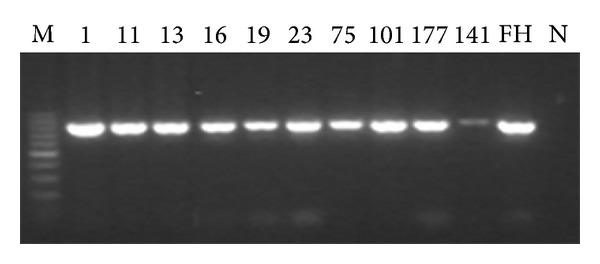
Electrophoretogram of the partial PCR products domain II of 23S rRNA of the strain (816 bp).

**Figure 2:**
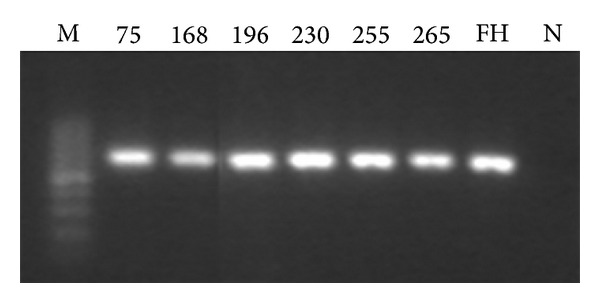
Electrophoretogram of the partial PCR products of the strain's ribosomal protein L22 (627 bp).

**Figure 3:**
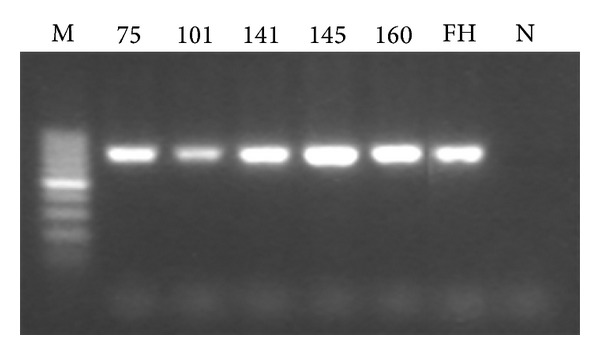
Electrophoretogram of the partial PCR products of the strain's ribosomal protein L4 (722 bp).

**Figure 4:**
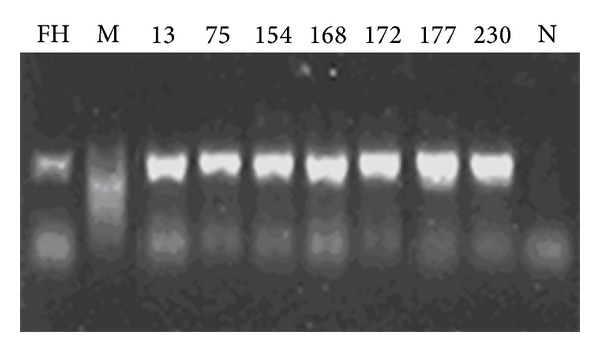
Electrophoretogram of the partial PCR products domain Vof 23S rRNA of the strain (793 bp).

Electrophoretograms of the four PCR products of genes targeted in this study are shown in Figures 1, 2, 3, and 4 (note: M represents the standard band, FH represents MP standard strain, N is the negative control, and digits refer to the strain number).

DNA sequencing results are shown in Table 2.

**Figure 5:**
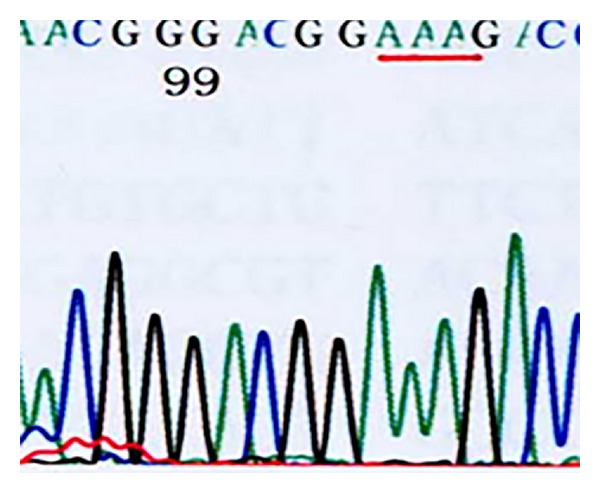
FH (no site mutation).

**Figure 6:**
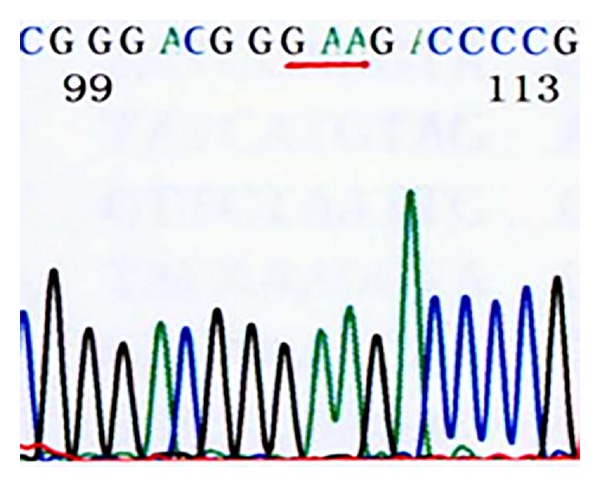
Clinically isolated MP strains (A2063G site mutation).

**Figure 7:**
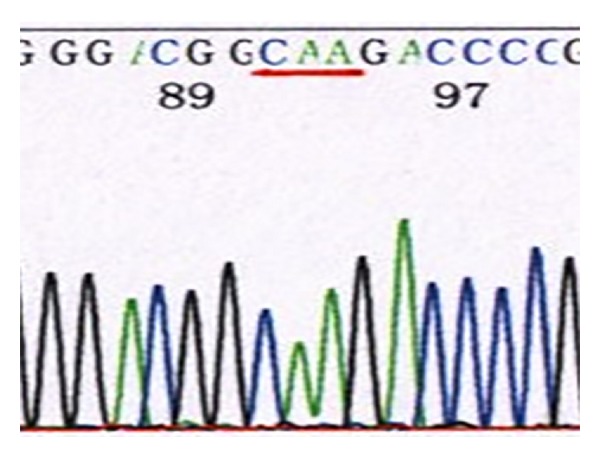
Clinically isolated MP strains (A2063C site mutation).

**Figure 8:**
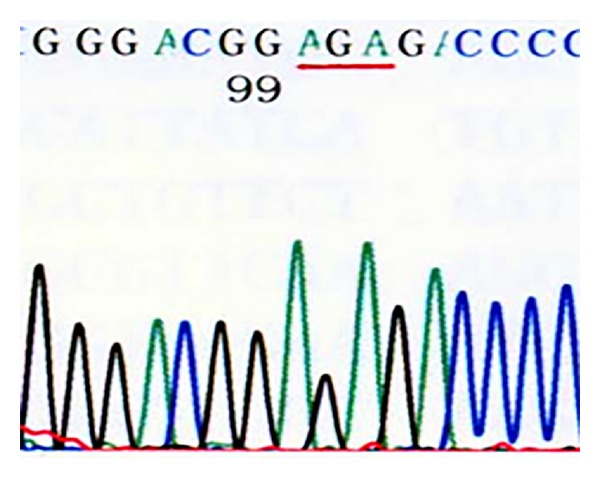
Clinically isolated MP strains (A2064G site mutation).

*Domain V of 23S rRNA*. 46 of the 50 previously cultured strains tested were resistant [11], and 40 of these strains showed the A2063G mutation, one strain showed an A2063C mutation, and five showed an A2064G mutation. Of the 26 recently cultured *M. pneumoniae* strains, 24 were resistant strains and they all showed the A2063G mutation. Six sensitive strains and the standard strain FH showed no mutations in this domain (Figures 5, 6, 7, and 8) (note: the part with a red underline in Figure 5 refers to the bases in the target sites 2063, 2064, and 2065 of the standard strain FH's 23S rRNA acted by erythromycin, all were A; Figure 6 represents 2063 site's base mutation A→G; Figure 7 represented 2063 site's base mutation A→C; Figure 8 represented 2064 site's base mutation A→G).

*Domain II of 23S rRNA*. 76 isolated *M. pneumoniae* strains and the standard strain FH all showed no gene mutation in domain II of 23S rRNA.

*Ribosomal Protein L4*. Of the 76 strains, 68 showed L4 gene fragments that were identical to M129, five strains and the standard FH showed C162A and/or A430G mutations, and five strains each showed one mutation in another site, namely C58A, T66G, G81T, A140C, and A209T.

*Ribosomal Protein L22*. With the exception of M129, all 76 isolated strains and the standard strain FH showed the T508C mutation. Five strains plus the standard FH strain also showed a T279C mutation and eight showed C62A and/or a T65A mutation.

Blast (http://blast.ncbi.nlm.nih.gov/) was used to detect the changes of amino acids encoded by the gene mutations of ribosomal proteins L4 and L22 (Table 3).

## 4. Discussion

The emergence of drug resistant *M. pneumoniae* strains is seriously reducing the effectiveness of macrolide drugs and affecting clinical outcome of *M. pneumoniae* infection in children [2, 7]. Before the year 2000, very few macrolide-resistant *M. pneumoniae* strains were isolated [7]. By contrast, since 2001, macrolide-resistant *M. pneumoniae* isolates were first reported by Japan [10], the frequency of macrolide-resistant *M. pneumoniae* cases has increased annually in Japan: 5.0% in 2003, 30.6% in 2006, 59.1% in 2009, and 89.5% in 2011 [12]. Similarly, since our team firstly reported the appearance of resistant strains of *M. pneumoniae* in 2005 [13], the frequency of macrolide-resistant *M. pneumoniae* remained high in China, ranging from 84.4% to 100% (not published) in our lab as well as in other Chinese researchers [9, 14]. In France, Pereyre et al. reported the emergence of *M. pneumoniae* drug resistant strains; only 2 of 155 showed resistance to macrolide isolated between 1994 and 2006 [11] but risen to 10% between 2005 and 2007 reported by Peuchant et al. [15]. In the other countries, the results were as follows: In USA, the frequency of macrolide-resistant *M. pneumoniae* was 8.2% during 2007 and 2010 [16], and in Italy that was 11 out of 43 in 2010 [17]; in Germany, 2 of 167 throat swabs were macrolide resistant collected between 2003 and 2008, reported by Dumke et al. in 2010, while during 1991 and 2009, only 3 of 99 isolation showed resistance [6], and so on. In conclusion, *M. pneumoniae* shows high resistance to macrolide antibiotics, especially in Asian, and it is rising annually, which should be taken into consideration by the world.

Macrolide antibiotics act by inhibiting bacterial protein synthesis. Studies have found that the target site for macrolide is the large (50S) subunit of the bacterial ribosome. Alterations in specific nucleotides within the 23S rRNA lead to decreased affinity between drug and ribosome. Many cases of macrolide resistance in clinical strains can be linked to mutations in the sites 2063, 2064, 2067, and 2617 in domain V of 23S rRNA [2, 7, 18]. In China, resistant strains have shown the 2063 and 2064 mutations but no mutations in any other sites [8, 9].

Ribosomal proteins L4 and L22 are also associated with drug resistance. These large subunit proteins have an elongated “tentacle” structure extending to the core of the large subunits to form the partial inner wall of the peptide output channel. Mutations within this structure can obstruct the channel and affect the binding of macrolide antibiotics [19]. In 2004, Pereyre et al. reported that amino acid changes of ribosomal proteins L4 and L22 could be induced *in vitro* appearing as H70R or H70L replacement, and 1~3 G insertion in site 60 in L4 as well as P112R and A114T replacement or _111_IPRA_114_ deletion in L22 [20]. However, no mutations were found in domain II of 23S rRNA in this study and it is not known whether the mutations induced *in vitro* arise in clinical samples [20].

In this study, among 76 clinical *M. pneumoniae* strains, 70 resistant strains were found showing A2063G, A2063C, and A2064G in domain V of 23S rRNA. None of the strains had any mutations in domain II of 23S rRNA. In addition to these major mutations, some of the strains showed C58A, T66G, G81T, A140C, and A209T mutations of L4, and C62A and T65A mutations of L22, further causing the changes of encoded amino acids. Whether there was a relationship between drug resistance and these mutations will require further study. C62A site mutation of ribosomal protein L22 was also observed in the erythromycin-induced strains, suggesting that there would be the possibility of inducing resistant strains *in vivo* during the process of using macrolide antibiotics.

Compared with M129, some strains, including the standard FH strain, showed C162A and A430G mutations of L4 and T279C and T508C mutations of L22 (Table 2). These were associated with two types of *M. pneumoniae* strains classified according to the difference in P1 gene, M129 being typical of P1-I type, and FH typical of P1-II type [21]. These mutations were therefore considered to be independent of *M. pneumoniae* drug resistance to macrolide. However, all the strains including the FH showed the T508C mutation in L22, and the P1 gene is considered to be independent of *M. pneumoniae* resistance to macrolide, T66G mutation in the ribosomal protein L4 of strain number 221 was synonymous and considered to be unrelated to drug resistance. A140C mutation in L4 of the MEN30 strain causes a Q47P change, but as MEN30 is a sensitive strain, it is inferred that this change is independent of resistance to macrolide. The A209T mutation in the ribosomal protein L4 of number 371 strain causes a H70L change. Pereyre et al. reported in 2004 that the telithromycin-induced *M. pneumoniae* resistant strain (T32) also showed a H70L change in L4, the mutation of C2617A in 23S rRNA domain V, and a A114T change in L22 [20]. Meanwhile, strain No. 371, a sensitive strain, showed a 2617 site mutation in 23S rRNA domain's zone V which is directly related to drug resistance as well as to changes in L4. Therefore, it is thought that the H70L change in L4 is unlikely to be related to drug resistance, although this needs further investigation. The remaining strains showed C58A and G81T site mutations in L4 as well as C62A and T65A site mutations in L22. Although both cause amino acid changes, but they also have the 2063 or 2064 mutation in the 23S rRNA domain V which is directly related to macrolide resistance (Table 2). Further study is needed to ascertain whether these mutations are associated with drug resistance. In this study, some of the clinically isolated strains showed a C62A mutation in the ribosomal protein L22 also found by Matsuoka et al. in Japan [22], suggesting that the use of macrolide drugs is likely to induce *M. pneumoniae* resistant strains.

*In vitro* drug sensitivity tests proved that the majority of children from whom the specimens were collected were harboring macrolide-resistant *M. pneumoniae* resistant strains. Some of the infection in these children could be controlled with macrolide antibiotics, mainly because macrolide antibiotics have an antimicrobial effect *in vivo* and are also involved in immune regulation during the recovery process of the disease, giving some clinical effectiveness [22]. At the same time, *M. pneumoniae* pneumonia is considered to be a self-limiting disease. In this study, all specimens were collected from children in the wards, and most of them had been exposed to macrolide antibiotics before sampling. Sensitive bacteria would have been inhibited, and total bacterial load reduced. This obviously made it difficult to estimate the actual levels of resistance found in our patients. However, resistant *M. pneumoniae* was obviously present and suggests the necessary to be very vigilant in monitoring the phenomenon of *M. pneumoniae* resistance to macrolide antibiotics.

In this study, *in vitro* drug sensitivity tests were used to determine the antibacterial activities of tetracycline, ciprofloxacin, and gentamicin against *M. pneumoniae*. Tetracycline mainly inhibits bacterial protein synthesis, by binding the ribosomal 30S subunits and blocking the extension or hindering the release of the protein synthesized peptide chain. Ciprofloxacin acts on *M. pneumoniae* primarily through inhibition of its DNA gyrase, thus affecting DNA replication, transcription, and expression. Gentamicin is a type of aminoglycoside antibiotic that acts on ribosomal 30S subunits in bacteria, inhibiting the protein synthesis and damaging the integrity of the cell membrane, but it has a limited inhibitory effect on *M. pneumoniae*. In this study, we found that 2063 and 2064 mutations in domain V of 23S rRNA of 50S subunits did not affect the binding of tetracycline, ciprofloxacin, and gentamicin with *M. pneumoniae* and did not give rise to *M. pneumoniae* resistance to these drugs. The results showed that tetracycline and ciprofloxacin were generally effective on *M. pneumoniae*, and gentamicin was effective on the majority of *M. pneumoniae* strains, although it did have poor antibacterial activity against some of the *M. pneumoniae* strains.

Because there is a risk of adverse reactions with tetracycline, and the safety of ciprofloxacin patients below the age of 18 has not yet been established, neither of these drugs is suitable for use in children. Current research should therefore be focused on finding new tetracycline or quinolone drugs with a strong antibacterial activity but low side effects, so as to provide effective alternative choice of drugs for the treatment of resistant *M. pneumoniae* infections.

In summary, 76 *M. pneumoniae* strains were isolated and cultured, 70 of which were macrolide-resistant, being highly resistant to erythromycin and showing the 2063 and 2064 mutations in domain V of 23S rRNA which are the main genetic markers of drug resistance. The clinical *M. pneumoniae* strains and erythromycin *in vitro* induced *M. pneumoniae* resistant strains showed gene mutations in the same sites of the ribosomal protein L22, suggesting the possibility of inducing *M. pneumoniae* resistant strains *in vivo* during the use of macrolide drugs. These results indicate the importance of continued research on the resistance mechanisms of drug resistance and in particular an increased urgency in finding effective new antibacterial drugs suitable for treating *M. pneumoniae* in children.

## Figures and Tables

**Table 1:** Primer sequences and target fragment length of PCR amplification.

Primer name	Primer sequence (5′-3′)	Target fragment
23S rRNA II zone	Forward: AGTACCGTGAGGGAAAGGTG Reverse: TCCCAAGCGTTACTCATGCC	816 bp

Ribosomal protein L4	Forward: AAAAGCAGCACCAGTTGTAG Reverse: GGTTAGAACTGGTTTTAGCA	722 bp

Ribosomal protein L22	Forward: GTACATAACGGCAAGACCTT Reverse: GCAAGCCGTTGGAGTTTACT	627 bp

23S rRNAV zone	External P1: GCAGTGAAGAAGAACGAGGGG P2: CACACTTAGATGCTTTCAGCG	1012 bp

**Table 2:** Sequencing results of a part of clinically isolated strains.

Specimen number	Ribosomal protein L4							Ribosomal protein L22				23S ribosomal RNA	
	58	66	81	140	162	209	430	62	65	279	508	Domain II	Domain V
N129	C	T	G	A	C	A	A	C	T	T	T	—	—
FH	—	—	—	—	C→A	—	A→G	—	—	T→C	T→C	—	—
1	—	—	—	—	C→A	—	A→G	—	—	T→C	T→C	—	A2064G
19	—	—	—	—	C→A	—	A→G	—	—	T→C	T→C	—	A2063G
75	—	—	G→T	—	—	—	—	—	—	—	—	—	A2064G
147	—	—	—	—	—	—	—	C→A	—	—	—	—	A2063G
216	—	—	—	—	—	—	—	—	T→A	—	—	—	A2063G
221	—	T→G	—	—	—	—	—	—	—	—	—	—	A2063G
223	—	—	—	—	—	—	—	C→A	T→A	—	—	—	A2063G
246	C→A	—	—	—	—	—	—	—	—	—	—	—	A2063G
255	—	—	—	—	—	—	—	—	T→A	—	—	—	A2063G
262	—	—	—	—	—	—	—	C→A	T→A	—	—	—	A2063G
277	—	—	—	—	—	—	—	C→A	T→A	—	—	—	A2063G
340	—	—	—	—	—	—	—	—	T→A	—	—	—	A2063G
355	—	—	—	—	—	—	—	—	T→A	—	—	—	A2063G
371	—	—	—	—	—	A→T	—	—	—	—	—	—	—
MEN20	—	—	—	—	C→A	—	A→G	—	—	T→C	—	—	—
MEN29	—	—	—	—	C→A	—	A→G	—	—	T→C	—	—	—
MEN30	—	—	—	A→C	C→A	—	A→G	—	—	T→C	—	—	—

Note: — represented no base mutation by comparing with the corresponding sequences of M129 that NCBI has registered; 1, 19, 75, 147, 216, 221, 223, 246, 255, 262, 277, 340, and 355 were all clinically isolated resistant strains, while 371, MEN 20, MEN 29, and MEN 30 were the sensitive strains. M129 and FH were the standard strains and MP gene sequences registered in NCBI gene bank were based on M129.

**Table 3:** Amino acid changes of ribosomal proteins L4 and L22.

Specimen number	L4	L22	23S rRNA domain V
75	K27N	—	A2064G
147	—	P21Q	A2063G
216	—	L22Q	A2063G
221	—	—	A2063G
223	—	P21Q, L22Q	A2063G
246	L201	—	A2063G
255	—	L22Q	A2063G
262	—	P21Q, L22Q	A2063G
277	—	P21Q, L22Q	A2063G
340	—	L22Q	A2063G
355	—	L22Q	A2063G
371	H70L	—	—
30	Q47P	—	—

Note: — represented no change by comparing with the corresponding sequences of M129 that NCBI has registered. MP gene sequences registered in NCBI gene bank were based on M129.
